# Toward a Macaque Model of HIV-1 Infection: Roadblocks, Progress, and Future Strategies

**DOI:** 10.3389/fmicb.2020.00882

**Published:** 2020-05-13

**Authors:** Rajesh Thippeshappa, Jason T. Kimata, Deepak Kaushal

**Affiliations:** ^1^Southwest National Primate Research Center, Texas Biomedical Research Institute, San Antonio, TX, United States; ^2^Department of Molecular Virology and Microbiology, Baylor College of Medicine, Houston, TX, United States

**Keywords:** HIV-1, HSIV, stHIV, macaque model, pigtailed macaques, interferon, cross-species transmission, innate restriction

## Abstract

The human-specific tropism of Human Immunodeficiency Virus Type 1 (HIV-1) has complicated the development of a macaque model of HIV-1 infection/AIDS that is suitable for preclinical evaluation of vaccines and novel treatment strategies. Several innate retroviral restriction factors, such as APOBEC3 family of proteins, TRIM5α, BST2, and SAMHD1, that prevent HIV-1 replication have been identified in macaque cells. Accessory proteins expressed by Simian Immunodeficiency virus (SIV) such as viral infectivity factor (Vif), viral protein X (Vpx), viral protein R (Vpr), and negative factor (Nef) have been shown to play key roles in overcoming these restriction factors in macaque cells. Thus, substituting HIV-1 accessory genes with those from SIV may enable HIV-1 replication in macaques. We and others have constructed macaque-tropic HIV-1 derivatives [also called simian-tropic HIV-1 (stHIV-1) or Human-Simian Immunodeficiency Virus (HSIV)] carrying SIV *vif* to overcome APOBEC3 family proteins. Additional modifications to HIV-1 *gag* in some of the macaque-tropic HIV-1 have also been done to overcome TRIM5α restriction in rhesus and cynomolgus macaques. Although these viruses replicate persistently in macaque species, they do not result in CD4 depletion. Thus, these studies suggest that additional blocks to HIV-1 replication exist in macaques that prevent high-level viral replication. Furthermore, serial animal-to-animal passaging of macaque-tropic HIV-1 *in vivo* has not resulted in pathogenic variants that cause AIDS in immunocompetent macaques. In this review, we discuss recent developments made toward developing macaque model of HIV-1 infection.

## Introduction

The inefficient replication of HIV-1 in macaques has complicated the development of a true HIV-based animal model of AIDS. Alternative animal models such as infection of macaques with Simian Human Immunodeficiency Virus (SIV) or Simian-Human Immunodeficiency Virus (SHIV) containing HIV envelope (Env) or reverse transcriptase have been developed. These animal models have been extremely useful in understanding HIV pathogenesis and disease progression, as well as understanding the efficacy of vaccines and drugs. However, these models do have short comings. A major concern is the genetic difference between HIV-1 and SIV. These viruses are not likely to share immunodominant cytotoxic T-cell (CTL) epitopes, and the structural differences in Env protein may lead to qualitative differences in the antibody responses. Furthermore, although the SHIV-model allows for testing of HIV Env based vaccines, it is not possible to test vaccines or antiretroviral drugs targeting multiple HIV-1 proteins. For example, efficacy of vaccine approaches using HIV Gag and Nef as immunogens cannot be tested using SHIV as a challenge virus. Additionally, some of the vaccine candidates that showed protection against SHIV did not exhibit protective efficacy in human clinical trials. It is therefore important to rationally and minimally modify HIV-1 such that it can replicate and cause AIDS in macaque species. Such an animal model will be an extremely valuable tool for preclinical evaluation of vaccines and novel therapeutics. Therefore, there is a need for developing pathogenic HIV-1 variants that can cause AIDS in macaque species.

## Retroviral Restriction Factors

The four major retroviral restriction factors are the apolipoprotein B mRNA editing enzyme catalytic polypeptide 3 (APOBEC3 or A3) family of proteins, tripartite motif containing (TRIM) family of proteins, BST2/CD317/Tetherin, and sterile alpha motif (SAM) and histidine/aspartic acid (HD) domain containing protein 1 (SAMHD1) [reviewed in Thippeshappa et al. (2012); Saito and Akari (2013)]. They inhibit retroviral replication at different stages of the life cycle. The fact that each of these retroviral restriction factors are upregulated by type 1 interferons (IFN-1) and each gene has been under strong positive selection in non-human primates and humans in response to lentiviral infections, suggests the importance of these restriction factors in controlling infection (Sawyer et al., 2004; Liu et al., 2005; Sawyer et al., 2005; McNatt et al., 2009; Laguette et al., 2012; Lim et al., 2012; Zhang et al., 2012). Both HIV-1 and SIV has counter measures to overcome these restriction in humans, but not in other species, suggesting the importance of overcoming these restriction factors for cross-species transmission (CST). Here, we briefly describe the importance of overcoming these restriction factors for generation of macaque-tropic HIV-1 and its derivatives.

The APOBEC3 family proteins are cytidine deaminases that render Vif deficient HIV-1 progeny virions non-infectious in non-permissive cells but not permissive cells (Sheehy et al., 2002; Aghokeng et al., 2010). In the absence of Vif, APOBEC3G (A3G) is efficiently packaged into HIV-1 progeny virions, while only small amount of A3G is packaged into wild-type HIV-1, which results in induction of G to A mutations in the viral genome (Sheehy et al., 2003). Interestingly, Vif counteraction of A3G occurs in a species-specific manner (Mariani et al., 2003; Bogerd et al., 2004; Mangeat et al., 2004; Schrofelbauer et al., 2004; Xu et al., 2004). HIV-1 Vif protein can antagonize human A3G (hA3G) but not rhesus macaque (RM, *Macaca mulatta*) A3G (Mariani et al., 2003). Interestingly, RM A3G is antagonized by both the SIVmac and SIVagm Vif proteins (Mariani et al., 2003), suggesting that Vif-mediated inhibition of macaque APOBEC3 family proteins could be important for CST of HIV-1 to macaques.

TRIM5α was identified by screening a rhesus macaque complementary DNA (cDNA) expression library for genes that would block HIV-1 infection in human cells (Stremlau et al., 2004). In New World monkey cells, such as Owl monkeys (OWM, *Aotus trivirgatus*), a novel TRIM5-cyclophilin A fusion protein (TRIMcyp) exhibits a post-entry barrier to HIV-1 infection (Nisole et al., 2004; Sayah et al., 2004). Prevalence of TRIMcyp has also been found in pigtailed macaques (PTM, *Macaca nemestrina*), cynomolgus macaques (CM, *Macaca fascicularis*) and Indian origin RMs (Brennan et al., 2007; Liao et al., 2007; Brennan et al., 2008; Newman et al., 2008; Wilson et al., 2008; Kuang et al., 2009; Dietrich et al., 2011). Both TRIM5α and TRIMcyp proteins exhibit species-specific restriction. Human TRIM5α does not restrict HIV-1; however, both RM and CM TRIM5α restrict HIV-1 but not SIVmac (Hatziioannou et al., 2004; Stremlau et al., 2004; Nakayama et al., 2005). TRIMcyp proteins also exhibit a diverse range of restrictions. OWM TRIMcyp interferes with HIV-1 but not SIVmac (Nisole et al., 2004; Sayah et al., 2004), whereas RM TRIMcyp restricts HIV-2 and SIVagm but does not inhibit HIV-1 and SIVmac (Virgen et al., 2008; Wilson et al., 2008). Interestingly, TRIMcyp protein expressed by PTM and CM do not block either HIV-1 or SIVmac239 infection (Brennan et al., 2008). However, PTM TRIMcyp can restrict FIV, HIV-2 and SIVagm infection (Virgen et al., 2008). These studies suggest that both the TRIM5α and TRIMcyp proteins can act as barriers for CST of lentiviruses.

BST2 or Tetherin is an interferon (IFN) inducible, Vpu sensitive factor that interferes with the release of HIV-1 progeny virions from the cell surface (Neil et al., 2008; Van Damme et al., 2008). HIV-1 Vpu can antagonize human and chimpanzee BST2, but cannot overcome the activity of BST2 from RM, AGM, and mustached monkeys (*Cercopithecus cephus*) (Goffinet et al., 2009; Gupta et al., 2009; Jia et al., 2009; Lim and Emerman, 2009; McNatt et al., 2009; Rong et al., 2009). However, Vpu may not be absolutely required for replication in humans. This is due to the fact that there are Vpu sequences (derived from primary isolates or directly from patients) in the HIV database that contain a mutation in the start codon (Li et al., 1991; Theodore et al., 1996; Schubert et al., 1999; Dejucq et al., 2000). Interestingly, SIV_mac__239_ Nef can target RM, PTM, and AGM BST2 but cannot antagonize human BST2 (Jia et al., 2009; Zhang et al., 2009; Yang et al., 2010). Thus, although BST2 antagonism may not be absolutely required for HIV-1 infection in humans, counteraction of BST2 function may be required for CST of HIV-1 to macaques.

Sterile alpha motif (SAM) and histidine/aspartic acid (HD) domain containing protein 1 (SAMHD1) was identified as a novel HIV-1 restriction factor in myeloid cells by two independent groups. They showed that Vpx interacts with SAMHD1 and results in proteasome-mediated degradation in myeloid cells (Hrecka et al., 2011; Laguette et al., 2011), which was later found to be active in quiescent T cells as well (Baldauf et al., 2012; Descours et al., 2012). It is interesting that HIV-1 does not have a mechanism to overcome human SAMHD1 function. However, human and gibbon SAMHD1 can be degraded by Vpx proteins from HIV-2rod, SIVmac, and SIVsm but not by Vpx from SIVrcm and SIVmnd2. Interestingly, these Vpx proteins can induce degradation of rhesus macaque and mangabey SAMHD1 (Laguette et al., 2012). Thus, Vpx-mediated degradation of macaque SAMHD1 may be required for adaptation of HIV-1 to macaque species. In addition, importance of Vpx expression for SIV replication has also been noticed in CD4 T cells and activated PBMCs where SAMHD1 restriction is inactive (Guyader et al., 1989; Kappes et al., 1991; Yu et al., 1991; Akari et al., 1992; Belshan et al., 2012; Shingai et al., 2015). Interestingly, Vpx protein from SIVrcm and SIVmnd2 enhanced replication of HIV-1 in resting CD4 T cells in a SAMHD1-independent manner, suggesting that Vpx overcomes another restriction factor in CD4 T cells (Baldauf et al., 2017). Two recent studies identified the human silencing hub (HUSH) epigenetic repressor complex as a potential restriction factor that controls viral expression and is antagonized by Vpx (Chougui et al., 2018; Yurkovetskiy et al., 2018). Vpx associated with the HUSH complex and resulted in proteasome-mediated degradation (Chougui et al., 2018; Yurkovetskiy et al., 2018). Thus, Vpx-mediated antagonization of HUSH complex may also be required for efficient replication in macaque lymphocytes.

## Additional Resistance Factors

There is growing evidence for the existence of additional IFN stimulated genes (ISGs) that control HIV-1 replication. *In vitro*, IFN treatment has been shown to potently inhibit HIV and SIV replication (Ho et al., 1985; Kornbluth et al., 1989; Shirazi and Pitha, 1992; Meylan et al., 1993; Cheney and McKnight, 2010; Goujon and Malim, 2010). Several resistance factors have been identified using *in vitro* cell culture models that potently inhibit retroviral replication. Interestingly, how HIV-1 evades the function of these resistance factors is not clear. (1) Myxovirus resistance 2 (MX2) was identified, using cDNA screens as a factor that inhibits viral cDNA accumulation and integration in IFN-treated cells (Goujon et al., 2013; Kane et al., 2013; Liu Z. et al., 2013). MX2 has been shown to interact with viral capsid protein (Goujon et al., 2013; Kane et al., 2013; Liu S.Y. et al., 2013; Fribourgh et al., 2014; Fricke et al., 2014) and may prevent viral cDNA from entering the nucleus by mechanisms that are not clear. (2) Interferon-inducible transmembrane proteins (IFITMs), particularly IFITM1, IFITM2, and IFITM3, are type II transmembrane proteins found in various cellular membranes (Bailey et al., 2013; Li et al., 2013; Jia et al., 2014; Li et al., 2015). IFITMs restrict a number of enveloped viruses including HIV-1 (Lu et al., 2011; Yu et al., 2015; Tartour et al., 2017). They have been shown to block virus entry by impairing the hemifusion process (Li et al., 2013). IFITM proteins protect target cells from incoming virus by affecting virus-cell fusion and targeting virions to endosomal or lysosomal compartments (Weidner et al., 2010; Desai et al., 2014; Spence et al., 2019). IFITM proteins also incorporate in to the nascent HIV particles during virus assembly and decrease the infectivity of the virions (Compton et al., 2014; Tartour et al., 2014). (3) Another ISG that inhibits HIV-1 infection is schlafen (SLFN11), which inhibits virion production by affecting protein synthesis (Li et al., 2012). (4) Cholesterol-25-hydoxylase (CH25H) is an enzyme that converts cholesterol to 25-hyroxycholesterol (25-HC). Treatment of cultured cells with 25-HC has been shown to inhibit replication of several enveloped viruses, including HIV, by impairing fusion of viral envelope with cell membrane (Liu S.Y. et al., 2013; Gomes et al., 2018). (5) Zinc-finger antiviral protein (ZAP) is another ISG that inhibits HIV-1 replication in overexpressed cells by translational repression and viral mRNA degradation through recruitment of cellular mRNA degradation machinery (Zhu et al., 2011; Zhu et al., 2012). (6) Guanylate-binding protein 5 is a member of the IFN-inducible guanosine triphosphatase (GTPases) superfamily that inhibits HIV-1 infectivity by interfering with the N-linked oligosaccharide glycosylation modifications of the HIV envelope glycoprotein (Krapp et al., 2016; Hotter et al., 2017). This increases the incorporation of unprocessed immature gp160 into progeny virions resulting in decrease in infectivity of the virions (Hotter et al., 2017). (7) Interferon-stimulated gene 15 (ISG15) is a 15 kDa protein belonging to the family of ubiquitin-like modifiers. The conjugation of ISG15 to target proteins is called ISGylation. HECT and RLD domain containing E3 ubiquitin protein ligase 5 (HERC5) mediated ISGylation results in the in the accumulation of Gag at the plasma membrane (Wong et al., 2006; Woods et al., 2011). Furthermore, ISGylation inhibits ubiquitination of Gag and Tsg101, which is a protein involved in endosomal sorting complexes required for transport (ESCRT) pathway. So, inhibition of this interaction prevents HIV-1 release (Okumura et al., 2006; Pincetic et al., 2010).

## Non-IFN Induced Restriction Factors

Apart from IFN-inducible restriction factors, there are also constitutively expressed intrinsic restriction factors that inhibit HIV-1 replication. Serine Incorporator (SERINC) proteins are a class of proteins, comprising of five members (SERINC 1-5), that facilitate the incorporation of serines into membrane lipids (Inuzuka et al., 2005). Recently SERINC 3 and 5 were identified as inhibitors of HIV-1 infectivity that are counteracted by Nef (Rosa et al., 2015; Usami et al., 2015). In the absence of Nef expression, SERINC5 incorporates into budding virions and reduces the infectivity of virions through mechanisms that are not completely clear. HIV-1 Nef, as well as MLV GlycoGag and equine infectious anemia virus (EIAV) S2 proteins decrease the expression of SERINC5 at the plasma membrane and exclude them from virions, thus restoring the infectivity of virions (Pizzato et al., 2007; Usami et al., 2014; Rosa et al., 2015; Usami et al., 2015; Chande et al., 2016).

Another non-IFN induced restriction that inhibits HIV-1 release is The T cell Ig and mucin domain (TIM) protein (Li et al., 2019). Release of Nef-deficient HIV-1 is most potently inhibited by TIM1 compared to wild-type HIV-1. Interestingly, SERINC proteins enhance the ability of TIM-1 to block HIV-1 release likely by increasing TIM-1 expression at plasma membrane. Nef proteins of HIV-1 and other lentiviruses function as antagonists to overcome the TIM-mediated restriction in part by promoting TIM-1 internalization from the plasma membrane and sequestering TIM-1 within intracellular compartments (Li et al., 2019). Furthermore, MLV glycoGag and EIAV S2 proteins, which are known SERINC antagonists, can also relieve the ability of TIM-1 to block HIV-1 release, suggesting a role for SERINC proteins in TIM-mediated restriction (Li et al., 2019). This suggests that lentiviral Nef proteins counteract TIM-1 restriction in part through SERINC to facilitate HIV-1 release and replication.

## Adaptation of HIV-1 to Macaques: Lessons From Cross-Species Transmission of Lentiviruses to Humans

SIVs are present in more than 40 species of non-human primates (Apetrei et al., 2004; Sharp and Hahn, 2011). The ones most closely related to HIV-1 and HIV-2 were detected in chimpanzees and sooty mangabeys, respectively (Hirsch et al., 1989; Gao et al., 1999). The ability of lentiviruses to infect new species vary. For example, SIVcol (from colobus monkeys) is evolutionarily isolated, whereas SIVagm (from African green monkeys) frequently moves between host subspecies (Bell and Bedford, 2017). So far, only chimpanzees, gorillas, and sooty mangabeys have transmitted their viruses to humans. Four independent transmission events of SIVcpz and SIVgor from chimpanzees and gorillas to humans gave rise to HIV-1 groups M, N, O, and P (Gao et al., 1999; Sharp and Hahn, 2011; D’Arc et al., 2015). HIV-2 groups A through I arose from nine zoonotic transmission events of SIVsmm from sooty mangabeys to humans (Hirsch et al., 1989; Gao et al., 1992; Chen et al., 1996; Ayouba et al., 2013). Studies over the years have demonstrated the key viral adaptations that led to successful CST of HIV-1 precursors from monkeys to chimpanzees, and then ultimately to humans [reviewed in Sauter and Kirchhoff (2019)]. Early report by Bailes et al. (2003) suggested that SIVcpz resulted from a recombination between an ancestor of SIVs found in greater spot-nosed, mona, and mustached monkeys (SIVgsn/mon/mus) and a SIV precursor found in redcapped mangabeys (SIVrcm). However, recent phylogenetic analysis studies from Bell and Bedford (2017) suggest cross-species transmission of SIV from redcapped mangabeys to mandrills (Bell and Bedford, 2017). Thus, it is not clear if part of the SIVcpz genome originated from SIVrcm or SIVmnd2. There are still large portions of the SIVcpz genome with unknown origins. Bell and Bedford (2017) also suggest that the genome of SIVcpz may have resulted from recombination of SIVrcm, SIVgsn/mon/mus, and an unknown SIV.

For CST to happen, lentiviruses have to adapt to utilize a number of cellular virus-dependency factors required for replication in the host. In addition, lentiviruses have to develop mechanisms to evade or counteract a variety of intrinsic restriction factors at every step of the virus life cycle. Recent studies have provided clues as to how this hybrid virus adapted to utilize some of the host-dependency factors as well as counteraction mechanisms required for spread in chimpanzees. The inability of chimpanzee TRIM5α to restrict many SIVs, including SIVgsn (Kratovac et al., 2008), probably presented an advantage for transmission of SIV from monkeys to chimpanzees. Interestingly, the recombination event that created SIVcpz resulted in the deletion of *vpx* coding sequences and generation of a unique *vif* that overlaps with *vpr* (Etienne et al., 2013). Unlike most SIV Vif proteins, this Vif protein from SIVcpz can antagonize chimpanzee A3G and A3D (Etienne et al., 2013). Additionally, chimpanzee A3F and A3H did not constitute a major barrier as SIVrcm Vif can antagonize these restriction factors (Etienne et al., 2013). However, the deletion of *vpx* may have cost the ability of these viruses to antagonize SAMHD1 and there by affecting the ability of SIVcpz and HIV-1, to infect myeloid cells and resting CD4+T cells. For adaptation to new species, lentiviruses also have to overcome additional restriction factors such as SERINC and BST2 at the late stage in virus life cycle. HIV-1 and SIV Nef can counteract SERINC5 in a species-independent manner (Heigele et al., 2016). Thus, it did not constitute a major barrier. However, SIVcpz needed to evolve mechanism to antagonize chimpanzee BST2. SIVcpz obtained its *vpu* gene from SIVgsn/mus/mon lineage, which can counteract monkey but not chimpanzee BST2 (Sauter et al., 2009). Although origin of the *nef* gene in SIVcpz is not clear, it is either derived from the SIVrcm lineage or from an unknown SIV (Bell and Bedford, 2017). Interestingly, during adaptation, SIVcpz evolved *nef* as a mechanism to antagonize BST2, and Vpu retained its other functions such as ability to degrade CD4. Apart from overcoming restriction factors, adaptation of SIVcpz to chimpanzees also coincided with changes in Gag that enabled interaction with host dependency factor RanBP2/Nup358 (Meyerson et al., 2018). This interaction of capsid with the nuclear pore protein RanBP2/Nup358 is required for efficient nuclear import of the viral genome (Ocwieja et al., 2011).

Transmission from chimpanzees to gorillas occurred probably because the virus was able to maintain interactions with host dependency factors as well mechanisms to counteract restriction factors in gorillas. Furthermore, SIVgor also acquired mutations in *nef* and *vif* to overcome gorilla BST2 and A3G mediated species-specific barriers, respectively (Sauter et al., 2009; Letko et al., 2013; D’Arc et al., 2015). These additional modifications probably helped SIVgor to replicate efficiently in gorillas. Adaptation to chimpanzees and gorillas probably made it easier for successful transmission of both SIVcpz and SIVgor to humans. Both SIVcpz and SIVgor Vif can effectively antagonize human APOBEC3G, F, and D proteins (Etienne et al., 2013; Zhang et al., 2017). SIVcpz and SIVgor Nef proteins cannot antagonize human BST2 function due to the deletions of the cytoplasmic domain (Jia et al., 2009; Sauter et al., 2009; Zhang et al., 2009). However, Vpu from pandemic HIV-1 M strains can counteract human BST2 (Sauter et al., 2009). Interestingly, only a few changes in the transmembrane domain of SIVcpz Vpu result in the ability to antagonize BST2 (Lim et al., 2010; Vigan and Neil, 2010; Kluge et al., 2013). Ability to overcome these restriction factors probably helped HIV-1 adapt to humans.

CST of SIVsmm from sooty mangabeys to humans occurred on nine independent occasions and gave rise to HIV-2 groups A-I (Hirsch et al., 1989; Gao et al., 1992; Chen et al., 1996; Ayouba et al., 2013). This jumping of SIV from monkeys to chimpanzees and then to humans has provided an excellent opportunity to understand the events and adaptations that are required for CST. SIVsmm adaptation to humans may have been easier as these viruses can replicate efficiently in human PBMCs (Gautam et al., 2007). SIVsmm Vif can counteract APOBEC3G from many species including human APOBEC3G (Letko et al., 2013). Interestingly, SIVsmm uses both Nef and Env to counteract BST2 in its natural hosts, and Env protein to antagonize human BST2 (Heusinger et al., 2018). This ability to antagonize human BST2 may have facilitated zoonotic transmission of SIVsmm to human. Another advantage for SIVsmm is that its Vpx can counteract human SAMHD1 and HUSH complex (Laguette et al., 2012; Chougui et al., 2018). Furthermore, SIVsmm Nef can antagonize human SERINC5 (Heigele et al., 2016). This ability of SIVsmm accessory proteins to overcome the function of human restriction factors may have facilitated the zoonotic transmission of SIVsmm to humans. However, HIV-2 strains may not have adapted very well for replication in human hosts. HIV-2 strains show higher A3G/F induced hyper mutations compared to HIV-1 (Bertine et al., 2015), suggesting inefficient degradation of these restriction factor by the HIV-2 Vif protein. Similarly, HIV-2 capsid is more susceptible to inhibition by human TRIM5α (Ylinen et al., 2005; Takeuchi et al., 2013). HIV-2 is also less pathogenic in humans compared to HIV-1. In most infected individuals, HIV-2 viral loads are controlled and results in slow disease progression (Martinez-Steele et al., 2007; Nyamweya et al., 2013).

Overall, these studies demonstrate that adaptation to the human host requires the ability of these viruses to counteract interferon-inducible restriction factors and the ability to exploit cellular dependency factors for virus replication.

## Adaptation of Rationally Modified HIV-1 to Macaque Species

The counter measures used by primate lentiviruses have guided the rational modification of HIV-1 for replication in macaque species. These rationally modified HIV-1 with SIV gene substitutions are designated macaque-tropic HIV-1 (mtHIV-1) or simian-tropic HIV-1 (stHIV-1) or human-simian immunodeficiency virus HSIV (listed in Table 1). Since, HIV-1 cannot overcome RM TRIM5α and APOBEC3 family of restriction factors, a simian-tropic HIV-1 (stHIV-1) was initially developed by incorporating *capsid* and *vif* sequences from SIVmac239 (Hatziioannou et al., 2006). stHIV-1, whose genome is 88% HIV-1 derived, replicated robustly in a RM T-cell line and RM PBMCs after *in vitro* adaptation (Hatziioannou et al., 2006). This suggest that avoidance of capsid- and Vif-based restriction may be sufficient to allow cross-species transmission of HIV-1 to rhesus macaques. However, the replication efficiency of stHIV-1 *in vivo* is unknown.

**TABLE 1:** *In vivo* replication of macaque-tropic HIV-1 derivatives.

Macaque-tropic HIV-1 derivatives	*In vivo* replication
NL-DT5R: HIV-1 carrying SIVmac *vif* gene and a short 21 nucleotide segment from the SIV capsid sequence corresponding to the HIV-1 cylophilin A binding loop	2 CD8 MAb-treated PTMs: Peak viremia 3.5 × 10^4^ RNA; detectable viral loads maintained until 10 to 11 wpi Igarashi et al., 2007 2 untreated PTMs: Peak viremia 5.6 × 10^3^ copies/ml; viral loads undetectable by 5 wpi Igarashi et al., 2007 1 CM: Viral loads were marginal (around 10^3^ copies/ml) and disappeared at 4 wpi Saito et al., 2011
HIV-1mt ZA012: NL-DT5R with a CCR5-tropic subtype C env	2 PTMs: Peak viremia of 1 × 10^6^ and 2.3 × 10^6^ copies/ml: detectable viral loads up to 16 wpi in one of the infected PTM Otsuki et al., 2014
MN4-5S: NL-DT5R incorporated with mutations identified during long-term passaging in CM-derived HSC-F cells as well as insertion of the loop between alpha helices 6 and 7 (L6/7) of the SIVmac capsid	3 CMs: 10-fold higher peak viremia (around 10^4^ copies/ml) at 2–3 wpi than NL-DT5R infected CM; viremia undetectable at 6 wpi Saito et al., 2011
MN4Rh-3: MN4-5S with Q110D mutation on helix 6 in capsid	6 TRIMcyp homozygote CMs: Peak viremia ranging from 1.1 × 10^4^ to 1.5 × 10^5^ copies/ml (mean 4.2 × 10^4^ copies/ml) at 2-4 wpi; viremia disappeared by 6 to 8 wpi Saito et al., 2013 3 TRIM5α homozygote CMs: Peak viremia of 1.9 × 10^3^ copies/ml and became undetectable at 4 wpi Saito et al., 2013
CXCR4- tropic MN4/LSDQgtu: MN4Rh-3 with M94L/R98S/G114Q substitutions in capsid and transmembrane domain of SIVgsn166 *vpu*	2 RMs: Peak viremia of ∼10^5^ viral RNA copies and viral loads became undetectable at 5-6 wpi Doi et al., 2018
gtu+A4Cl1: MN5/LSDQgtu carrying *env* gene from clinical isolate	1 RM: Peak viremia around 10^4^ copies/ml and undetectable at 3-4 wpi Doi et al., 2018
stHIV-1: HIV-1 derivatives carrying either SIVmac *vif* or HIV-2 *vif*	4 PTMs: Peak viremia of 10^5^ to 10^6^ copies/ml; detectable viremia persisted for 25 wpi Hatziioannou et al., 2009
HSIV-vif: HIV-1 derivative carrying *vif* gene from highly pathogenic PTM-adapted SIVmne027	2 Juvenile PTMs: Plasma viral loads peaked (1.4 to 4.04 × 10^4^ viral RNA) at 2 wpi and showed extended viral replication through 44 wpi and small rebounds in viral titer at 64 and 72 wpi Thippeshappa et al., 2011. 2 Newborn PTMs: Peak viremia of 0.5 × 10^5^ to 1.0 × 10^5^ vRNA copies/ml; rapidly declined and below the limit of detection within 8 to 20 wpi; modest rebound in viral loads between 100 and 1,000 vRNA copies/ml around 24 wpi Thippeshappa et al., 2011
stHIV-1 carrying CCR5-tropic env from YU2, BaL, AD8, and KB9.	2 PTMs: peak viremia around 10^5^ copies/ml and one of the infected macaques maintained viral loads of 10^3^ copies/ml up to 32 wpi Hatziioannou et al., 2014
stHIV-A18+stHIV-A19: infectious molecular clones isolated from passage 4 PTM	2 PTMs: Peak viremia 6.1 × 10^5^ and 1.2 × 10^6^ copies/ml; detectable viral loads up to 100 to 150 wpi; gradual decline in CD4 T cell in one of the infected macaques Schmidt et al., 2019
stHIV-A19: infectious molecular clone isolated from passage 4 PTM	3 CD8 depleted PTMs: Peak viremia >10^6^ copies/ml; setpoint viral loads >10^5^ copies/ml; CD4 T cells depletion by 27 wpi Schmidt et al., 2019 1 untreated PTM: peak viremia >10^6^ copies/ml; persistence of 10^2^ to 10^3^ copies/ml up to 25 wpi Schmidt et al., 2019

To minimize the sequences from SIV, a variant HIV-1 which carries only the SIVmac *vif* gene and a short 21 nucleotide segment from the SIV capsid sequence corresponding to the HIV-1 cylophilin A binding loop has been constructed (Kamada et al., 2006). Long-term passaging of this clone in a CM lymphoid cell line resulted in an *in vitro* adapted HIV-1 derivative (NL-DT5R), which replicated well in the CM T-cell line (HSC-F) as well as CD8^+^ T-cells depleted T-cells from five of five PTMs and one of three RMs. To assess replicative and disease-inducing properties *in vivo*, 4 PTMs were inoculated intravenously with 1.9 × 10^6^ TCID_50_ of NL-DT5R virus generated from CD8 + T-cell-depleted pig-tailed macaque PBMC. Two of the PTMs were treated with anti-human CD8 monoclonal antibody (MAb) cM-T807 subcutaneously on day 1 (10 mg/kg of body weight), and intravenously on days 4, and 7 (5 mg/kg of body weight) post-infection. HIV-1 NL-DT5R established productive infections in all four animals with no substantial difference in the levels of peak viremia (5.6 × 10^3^ to 3.5 × 10^4^ RNA copies/ml) in the untreated and anti-CD8 MAb-treated monkeys. However, plasma viremia became undetectable by week 5 post-infection in the two untreated macaques, whereas viremia was maintained until weeks 10 to 11 in the two treated animals. Although NL-DT5R established a productive infection and elicited humoral responses against all of the HIV-1 structural proteins in PTMs, it did not cause CD4^+^ T cells depletion or disease (Igarashi et al., 2007). To further adapt NL-DT5R to PTMs, an additional macaque was inoculated intravenously with virus inoculum containing lymph node cells collected from each of the 4 monkeys (7.5 × 10^7^ cells) suspended in 20 ml of pooled whole blood. This animal was also transiently depleted of CD8 T cells by treating with the anti-CD8 MAb at the same doses and routes as two of the monkeys in the initial infection. The plasma viral loads in this PTM peaked (1.9 × 10^4^ RNA copies/ml) at week 2.4 post-infection and then rapidly declined, becoming undetectable at week 6 post-infection (Igarashi et al., 2007). A new macaque-tropic HIV-1 (named HIV-1mt ZA012) carrying *env* from a CCR5 tropic subtype C HIV-1 clinical isolate (HIV-1 97ZA012) in the back bone of NL-DT5R was generated by intracellular homologous recombination (Otsuki et al., 2014). To improve the replication competence, HIV-1mt ZA012 was serially passaged in PTM PBMCs. Virus supernatant from passage 19 replicated better than NL-DT5R and HIV-1mt ZA012-P0 in CD8 T cell depleted PTM PBMCs. To study the *in vivo* replication capacity, two PTMs were inoculated intravenously with passage 19 virus grown in PTM PBMCs. PTM1 showed peak viremia of 1 × 10^6^ copies/ml at week 2 post-infection and became undetectable by 8-week post-infection (wpi). PTM2 exhibited a peak viremia of 2.3 × 10^6^ copies/ml at 1.5 wpi, maintained viral loads of 10^4^ copies/ml, became undetectable at 16 wpi (Otsuki et al., 2014).

*In vivo* replication and disease-causing potential of NL-DT5R has also been studied in CMs. NL-DT5R established infection in CMs. However, viral loads were marginal and disappeared by week 4 post-infection (Saito et al., 2011). In order to improve the replication capability of NL-DT5R in CM, long-term passaging in CM-derived HSC-F cells were conducted. Additionally, NL-DT562 having CCR5-tropic *env* gene on a background of NL-DT5R was also passaged long-term in HSC-F cells. Long-term passaging improved the replication potential of both CXCR4 and CCR5 tropic NL-DT5R and resulted in a total of 14 mutations (10 in the NL-DT5R-derived clone and 4 in the NL-DT562-derived clone). These mutations were introduced into the parental NL-DT5R clone to generate a clone named MN4-5 (Saito et al., 2011). Previously, it was found that insertion of an SIVmac loop between alpha helices 6 and 7 (L6/7) of capsid into the corresponding region in HIV-1 significantly enhanced the NL-DT5R replication in HSC-F cells and PBMCs of CM by relieving the inhibitory effect of TRIM5α (Kuroishi et al., 2009). Therefore, A modified MN4-5 clone (named MN4-5S) was generated by inserting the loop between alpha helices 6 and 7 (L6/7) of the SIVmac capsid into the corresponding region in HIV-1 (Saito et al., 2011). MN4-5S showed enhanced replication compared to the parental NL-DT5R in CM-derived HSC-F cells and CD8^+^ T-cells depleted PBMCs from CMs. In intravenously (IV) inoculated CMs, MN4-5S resulted in 10-fold higher peak viremia at 2-3 wpi compared to NL-DT5R infected CMs. However, the viremia became undetectable at 6 wpi, partly due to control by CD8^+^ T-cells as *in vivo* depletion of CD8 + cells resulted in the reappearance of viremia (Saito et al., 2011).

MN4-5S has been further adapted in macaque cells by passaging and an adaptive mutation in capsid that enhances growth ability in the cells has been identified (Nomaguchi et al., 2013b). *In silico* structural modeling predicted that Q110D mutation on helix 6 in capsid (CA-Q110D) would promote viral replication in macaque cells. Therefore, a proviral clone carrying CA-Q110D, designated MN4Rh-3, was constructed. Indeed, MN4Rh-3 exhibited marked enhancement of growth potential in macaque cells relative to other mtHIV-1clones that have been constructed. Interestingly, the CA-Q110D mutation did not contribute to enhancement of further resistance to TRIMCyp or evasion from TRIM5α restriction (Nomaguchi et al., 2013b). To investigate whether *TRIM5* genotypes could influence the growth of MN4Rh-3 *in vivo*, viral stocks propagated in CD8^+^ cell-depleted PBMCs were inoculated intravenously into TRIMCyp homozygotes (*n* = 6) or TRIM5α homozygotes (*n* = 3) (Saito et al., 2013). MN4Rh-3 replicated readily in all TRIMCyp homozygotes, with plasma viral loads reaching a peak at 2–4 weeks post-inoculation and ranging from 1.1 × 10^4^ to 1.5 × 10^5^ copies/ml (mean 4.2 × 10^4^ copies/ml). In contrast, MN4Rh-3 replicated poorly in TRIM5α homozygotes (mean peak viremia 1.9 × 10^3^ copies/ml) (Saito et al., 2013). As expected, HIV-1-specific antibodies were detected in the TRIMCyp homozygotes but minimally in TRIM5α homozygotes suggesting that the strength of antibody response reflected the level of virus replication (Saito et al., 2013). To adapt MN4Rh-3 to RMs, *gag* and *vpu* were altered to overcome TRIM5α and BST2 function (Nomaguchi et al., 2013a). Using sequence- and structure-guided mutagenesis, three amino acid substitutions in capsid (M94L/R98S/G114Q) were introduced to overcome TRIM5α susceptibility. Additionally, transmembrane domain of *vpu* was replaced with the corresponding region of simian immunodeficiency virus SIVgsn166 *vpu*. The resultant clone, designated MN4/LSDQgtu, antagonized macaque but not human BST2, and replicated efficiently in a macaque cell line. Notably, MN4/LSDQgtu grew comparably to SIVmac239 and much better than other mtHIV-1clones in RM PBMCs (Nomaguchi et al., 2013a). A CCR5-tropic version of MN4/LSDQgtu carrying HIV-1 SF162 *env*, designated MN5/LSDQgtu (or 5gtu) has also been constructed. However, MN5/LSDQgtu replicated poorly compared to MN4/LSDQgtu in RM cell line (Doi et al., 2013). Additional CCR5-tropic clones carrying pSHIV_AD__8__–EH_ or *env* clones from clinical isolates have been generated in the backbone of MN5/LSDQgtu (Doi et al., 2017). Interestingly, two of the clones carrying *env* genes from clinical isolates, designated gtu + Cl1 and gtu + A4Cl1, grew better than the parental clone MN5/LSDQgtu (or 5gtu) in RM M1.3S cells. Furthermore, gtu+A4Cl1 grew comparably well with MN4/LSDQgtu in PBMCs isolated from two different donor RMs (Doi et al., 2017). To determine the replication potential *in vivo*, two RMs were challenged with CXCR4- tropic MN4/LSDQgtu and one RM with CCR5-tropic gtu+A4Cl1 (Doi et al., 2018). Although both viruses established infection in RMs, MN4/LSDQgtu replicated better with peak viremia of ∼10^5^ viral RNA copies compared to 10^4^ copies/ml for gtu+A4Cl1. However, virus replication was transient and became undetectable at 5–6 wpi (Doi et al., 2018).

## Adaptation of Minimally Modified HIV-1 to Ptms

Among non-human primates, PTMs are known to be uniquely susceptible to HIV-1 infection (Agy et al., 1992; Frumkin et al., 1993; Gartner et al., 1994a, b; Agy et al., 1997; Bosch et al., 1997; Bosch et al., 2000). Agy et al. (1992) first showed that PTMs can be infected with HIV-1. All the eight infected animals experienced sustained seroconversion to a broad range of HIV-1 proteins. Furthermore, virus could be recovered from the infected macaque PBMCs by co-cultivation and proviral sequences could be detected in DNA isolated from PBMCs. However, cell free virus was detected in the plasma of only one infected macaque (Agy et al., 1992). To accelerate adaptation of HIV-1 to PTMs, blood from infected macaques was serially transfused into three groups of naive macaques. At three to 5 weeks after transfusion, plasma viral loads from several macaques in the first two groups exceeded those of the initially inoculated macaques. Unexpectedly, animals in the third group had diminished RNA levels, virus culture negative, and did not seroconvert. It was later found out that the blood used for transfusion was virus-culture negative (Agy et al., 1997).

In another study, four PTMs were inoculated with autologous cells expressing low amounts of HIV-1 (Gartner et al., 1994a). Infectious virus could be recovered from PBMCs and lymph nodes up to 10 wpi in 3 out of 4 infected macaques. Further, HIV-1 DNA was frequently detected in uncultured PBMCs from all three animals. In one of the infected animals, virus could be re-isolated at 38- and 61-weeks post-infection, suggesting that the animal was persistently infected with HIV-1. Interestingly, *in vivo* passaging of the virus at week 6 post-infection did not select for pathogenic variants. One PTM and one CM that received transfusion of virus-positive blood and lymph node cells failed to become detectably infected (Gartner et al., 1994b). Attempts have also been made to adapt HIV-1 to newborn PTMs. In the case of neonate PTMs, three out of five rectally exposed and two of two intravenously inoculated macaques became infected with HIV-1. However, none of the four orally exposed animals showed evidence of HIV-1 infection. Although HIV-1 replicated more vigorously in newborns, passaging of HIV-1 in newborn PTMs did not result in the emergence of pathogenic variants capable of causing CD4 depletion (Bosch et al., 1997; Bosch et al., 2000). However, the long-term presence of HIV-1-specific antibodies, proviral sequences, and the recovery of infectious virus in these studies indicate the unique susceptibility of PTMs to HIV-1 infection.

We have also observed that PTM PBMCs can be more easily transduced with VSVG pseudotyped HIV-1 than RM PBMCs, suggesting the absence of a post-entry block (Thippeshappa et al., 2011). Several groups have observed the absence of the retroviral restriction factor, TRIM5α, in this macaque species. Moreover, novel isoforms of TRIM5 [TRIM5θ, which lacks B30.2 (SPRY) domain and TRIM5η, which has a deletion of the entire exon 7] expressed by PTMs do not restrict HIV-1 infection (Brennan et al., 2007). Interestingly, PTMs express a TRIM5-cyclophilin A fusion protein (TRIMcyp) due to LINE-mediated retrotransposition of the cyclophilin A cDNA into the untranslated region of exon 8 of the TRIM5 locus (Liao et al., 2007; Brennan et al., 2008; Newman et al., 2008; Virgen et al., 2008). However, unlike OWM TRIMcyp, the PTM TRIMcyp does not restrict HIV-1 infection (Liao et al., 2007; Brennan et al., 2008; Newman et al., 2008; Virgen et al., 2008).

Absence of TRIM5α presents an advantage for the development of a minimally modified HIV-1 that can potentially infect and cause AIDS in PTMs. Since Vif expressed by SIVmac and HIV-2 can degrade RM APOBEC3G, Hatziioannou et al. (2009) constructed HIV-1 derivatives carrying either SIVmac *vif* or HIV-2 *vif* in place of HIV-1 *vif*. Intravenous (IV) Inoculation of PTMs with an admixture of these two viruses resulted in acute infection, and viremia persisted for 25 wpi. However, infection was controlled thereafter and it did not result in CD4^+^T-cell depletion. We also constructed an Human-Simian Immunodeficiency Virus, named HSIV-vif_NL__4__–__3_ by substituting pNL4-3 *vif* with *vif* gene from highly pathogenic PTM-adapted SIVmne027 (Kimata et al., 1998; Kimata et al., 1999). IV inoculation of PTMs with HSIV-vif_NL__4__–__3_ virus, generated from transfecting 293T cells, extended viral replication through 44 wpi and small rebounds in viral titer at 64 and 72 wpi in juvenile PTMs (Thippeshappa et al., 2011). Furthermore, viral DNA could be detected in PBMCs upto 92 wpi, suggesting that the animals were persistently infected for nearly 2 years (Thippeshappa et al., 2011). Hatziannou et al. conducted animal-to-animal transfer to generate pathogenic variants [discussed in Kimata (2014)]. Passage 1 PTMs were IV inoculated with an inoculum containing four clonal HIV-1_NL__4__–__3_–derived viruses, each encoding a gp120 Env protein from a prototype HIV-1 strain that uses the CCR5 co-receptor (YU2, BaL, AD8, and KB9). Passage 2 to 4 PTMs were transiently depleted of their CD8 T-cells at the time of inoculation. The resulting virus from passage 4 only caused CD4 depletion in animals transiently depleted of CD8 T cells. However, immunocompetent macaques controlled the viral loads (Hatziioannou et al., 2014). Interestingly, HIV-1 Vpu acquired mutations during *in vivo* adaptation to antagonize PTM BST2. Furthermore, *in vivo* adaptation also led to changes in HIV Env that improved its ability to bind macaque CD4 (Hatziioannou et al., 2014). Recently, they also reported acquisition of amino acid changes in capsid that conferred partial resistance to PTM MX2 resistance factor (Schmidt et al., 2019). To recapitulate the phenotype observed with viral swarm from the blood of passage 4 animal, the group of Hatziioannou also generated several IMCs. One of the clones, named stHIV-A19, caused CD4 depletion only in macaques that were transiently depleted of CD8 T cells. However, it was controlled in immunocompetent PTMs (Schmidt et al., 2019). We also conducted animal-to-animal transfer of infected blood in 3 immunocompetent PTMs. Our initial inoculum contained a mixture of transfection supernatants of HSIV-vif_NL__4__–__3_, HSIV-vif_AD__8_, and HSIV-vif_YU__2_, and virus recovered from co-culture of PBMCs isolated from previously infected macaque and naïve CD4 T cells. However, the viral loads remained controlled in all 3 macaques (unpublished data).

## Possible Role of Interferon Response in Control of Macaque-Tropic HIV-1 Infection

The reasons for virologic control in the immunocompetent PTMs remain unclear. CD8^+^ T-cell depletion studies suggest that cellular immune responses may be limiting replication of the macaque-tropic HIV-1 clones (Hatziioannou et al., 2009). Additionally, the Type 1 IFN response induced during acute infection might restrict viral replication to a level that may prevent establishment of infection to achieve high peak viremia and set point viral loads. Indeed, IFNs are upregulated during HIV-1 and SIV infections (Bosinger et al., 2009; Jacquelin et al., 2009; Stacey et al., 2009; Sandler et al., 2014). Induction of IFNα results in the expression of many IFN-stimulated genes (ISGs) and the establishment of an antiviral state in the cell. Significantly, retroviral restriction factors (i.e. APOBEC3 family proteins, TRIM5α, BST2, and SAMHD1) are upregulated by IFN, linking restriction to the innate immune response [reviewed in Thippeshappa et al. (2012); Misra et al. (2013)]. Furthermore, IFN response also induces the expression of several resistance factors such as, myxovirus resistance 2 (MX2), IFN-induced transmembrane (IFITM) proteins, schlafen 11 (SLFN11), and other yet to be identified restriction factors that inhibit HIV-1 replication [reviewed in Doyle et al. (2015)]. Thus, it is possible that the IFN response during acute infection in PTMs restricts HIV-1 replication to a level that is not sufficient for adaptive mutations to occur. Therefore, Type 1 IFN response induced during acute infection suggests that these PTM-tropic HSIV-vif viruses should overcome the effect of restrictive ISGs in order to replicate to high levels and cause disease. We have previously demonstrated that the prototype PTM-tropic HSIV-vif_NL__4__–__3_ is inhibited by IFNα in PTM cells. However, pathogenic SIVmne and SIVmac clones resist IFNα-induced inhibition (Bitzegeio et al., 2013; Thippeshappa et al., 2013). Interestingly, we have identified a HSIV-vif derivative with YU2 Env that resists IFNα treatment in PTM CD4^+^ T cells. Using chimeric viruses between HSIV-vif_NL__4__–__3_ and HSIV-vif_YU__2_, we demonstrated that YU2 Env is the determinant that contributes to IFN-resistance. We also demonstrated, using Vpr-beta lactamase fusion assay, that HSIV-vif_YU__2_ overcomes IFN-induced restrictions at the entry step of the virus life cycle (Thippeshappa et al., 2013). However, further experiments need to be conducted to identify the IFN-induced restriction factors at the virion fusion step that restrict HSIV-vif_NL__4__–__3_ but not HSIV-vif_YU__2_.

Since many of the retroviral viral restriction factors are upregulated in the presence of IFN, it will be critical for the virus to overcome IFN responses to establish productive infection. Indeed, Transmitted/Founder (T/F) viruses have been shown to be IFN-resistant (Fenton-May et al., 2013; Iyer et al., 2017). Furthermore, adapting SHIVs to rhesus macaques (RMs) selects for Env-mediated IFNα-resistance (Boyd et al., 2016). Additionally, inhibition of the IFN-response by administration of a IFNα receptor antagonist has been shown to result in high viral loads during acute infection and faster progression to AIDS in a pathogenic SIV-RM model (Sandler et al., 2014). Thus, inoculating PTMs with IFN-resistant HSIV-vif may help to overcome the IFN response induced during acute infection leading to higher peak viremia. We have identified a variant PTM-tropic HSIV-vif_YU__2_ that resists IFNα-treatment in both human PBMCs and PTM CD4 T cells. Additionally, Vpx is essential for replication of SIV in macaques (Belshan et al., 2012; Shingai et al., 2015) and it enables the replication of SIV in non-dividing cells by overcoming SAMHD1 function (Hrecka et al., 2011; Laguette et al., 2011). SIV Nef has also been shown to inhibit the effects of macaque BST2 (Jia et al., 2009; Zhang et al., 2009). Therefore, additional modifications to the HSIV-vif_YU__2_ viral genome to overcome BST2-mediated and SAMHD1-mediated restriction may be required to establish persistent viremia *in vivo*. First generation SHIV constructs replicated poorly in macaques although long-term persistence was observed. Thus, serial *in vivo* passages were conducted to enhance the infectivity or replicative capacity of several SHIV strains (Luciw et al., 1995; Joag et al., 1996; Reimann et al., 1996; Igarashi et al., 1999; Chen et al., 2000; Song et al., 2006). We anticipate that serial *in vivo* passaging of IFNα-resistant HSIV-vif_YU__2_ or its derivatives carrying SIV *nef* or *vpx* genes will generate pathogenic variants with enhanced infectivity. However, a consequence of adapation to restriction factors may be fitness cost. Interestingly, a molecular clone with adapted capsid that showed complete resistance to PTM MX2 exhibited impaired replication capacity (Schmidt et al., 2019). This suggests that complete resistance to certain restriction factors may incur fitness defect to the virus. Therefore, adaptation to a new species may require acquisition of mutations to overcome restriction factors without incurring replication fitness cost.

## Conclusion

Inability of passaged viruses to cause AIDS in non-CD8 depleted macaques suggest that more work is required to develop a bon-a-fide macaque-model of HIV-1 infection. Overcoming initial IFN responses induced during acute infection may enable efficient replication of HSIV-vif in PTM host. Thus, we hypothesize that successful CST of HIV-1 to PTMs depends on inhibiting and evading IFN-induced restriction factors and that appropriate modifications to the genome will enable HIV-1 replication in the PTM host. We believe that infection of PTMs with IFN-resistant variants could provide insight into whether evasion of IFNα response is critical for viral replication in the host. Thus, IFN-resistant HSIV-vif viruses will be an excellent starting point for adaptation to PTMs and eventual development of macaque model of HIV-1 infection. Such an animal model would be extremely valuable for preclinical evaluation of novel vaccines and therapeutics, as these HSIV-vif clones have all the HIV immunologic and vaccine targets such as Gag, Env, Tat, Rev, and Nef. This is the drawback with SIV and SHIV models which are not ideal for testing vaccine approaches targeting HIV Gag and Nef antigens. Furthermore, establishment of HIV reservoirs in this model also provides an avenue for developing therapeutic vaccination approaches targeting HIV Gag and Env, apart from testing latency reversal agents. Once these models are established, they would also positively inform the process of refining models of HIV co-infections and co-morbidities, such as the *Mtb*/HIV co-infection model, which until now has relied on the use of SIV as a co-infecting agent in either rhesus (Mehra et al., 2011; Foreman et al., 2016; Bucsan et al., 2019) or cynomolgus macaques (Diedrich et al., 2010).

## Author Contributions

RT wrote the manuscript, while JK and DK provided guidance, technical support, inputs, and edited the manuscript.

## Conflict of Interest

The authors declare that the research was conducted in the absence of any commercial or financial relationships that could be construed as a potential conflict of interest.
